# Reviewed article: Paiva RFPS, Maia ALO, Martins JBC. Occurrences, human harm and years of life lost due to natural disasters in the state of Rio de Janeiro, Brazil, 2010-2022: a cohort study. Epidemiol Serv Saude. 2025;34:e20240412. 10.1590/S2237-96222025v34e20240412.en

**DOI:** 10.1590/S2237-96222025v34e20240412.b

**Published:** 2025-04-18

**Authors:** Elisa Prezotto Giordani

**Affiliations:** 1 Secretaria de estado de saúde do Espírito Santo, Espírito Santo, ES, Brasil

## Peer review B

## Questionnaire

Does the manuscript contain new and significant information to justify publication? Yes

Does the Abstract (Summary) clearly and accurately describe the content of the article? Yes

Is the problem significant and concisely stated? Yes

Are the methods described comprehensively? Yes

Are the interpretations and conclusions justified by the results? Yes

Is adequate reference made to other work in the field? Yes

## Manuscript Structure

Length of article is: Adequate

Number of tables is: Adequate

Number of figures is: Adequate

Please state any conflict(s) of interest that you have in relation to the review of this paper (state “none” if this is not applicable).

None

Interest: Excellent

Quality: Excellent

Originality: Excellent

Overall: Excellent

Recommendation: Minor Revision

## Comments

1. Indicar o tipo de estudo. No lugar de “estudo descritivo” usar “coorte prospectivo”.

2. No resumo, substituir “R$ 4 bi” por R$ 4 bilhões.

3. Conforme recomendação da revista, não utilizar termos em inglês que tenham correspondência em português. Remover “YLL” do texto.

4. Na tabela 3, suprimir a primeira linha ( já que há explicação no cabeçalho) e padronizar o número de casas decimais. Interessante deixar apenas os números inteiros para conferir aspecto mais limpo à tabela. Em sua última linha, o total apresentado causa confusão. Sugere-se uma terceira coluna para indicá-la.

5. O aspecto visual da tabela 1 seria beneficiado com a exclusão da coluna “mínimo” e a inclusão de observação sobre ela no cabeçalho.

